# Methods to find out the expression of activated genes

**DOI:** 10.1186/1477-7827-2-68

**Published:** 2004-09-23

**Authors:** Sten Z Cekan

**Affiliations:** 1 Karolinska Institute, Department of Woman and Child Health, Division of Reproductive Endocrinology, Karolinska University Hospital, Building L5, 17176 Stockholm, Sweden

## Abstract

This review deals with the methods of identifying genes that have been activated by inner or outer impulses. The activation and subsequent expression of a gene can be detected by its transcription into a corresponding messenger ribonucleic acid (mRNA). Principles of the methods for identification of individual activated genes, as well as groups of activated genes are described, the former methods being mostly based on subtractive hybridization and serial analysis of gene expression (SAGE), the latter on microarrays. Examples of gene activation by the hormone 17beta-estradiol (E2) are given.

## Introduction

In previous reviews, methods for the measurement of receptors and their interactions with other transcription factors and genes were described [1-3]. In this review, gene activation is discussed with a particular emphasis on the methods enabling detection of the activated, turned-on, genes. The action of the hormone 17beta-estradiol (E2) is taken as an example of the function of many other small-molecule compounds in gene activation and in the expression of the activated gene.

The life of humans and animals is influenced by the activity of a series of genes that are kept in a silent state, or are activated, depending on the temporary needs of the body. This switching on and off of each gene is executed by an assembly of transcription factors forming a transcription initiation complex (TIC).

Examples of such transcription factors are estrogen receptors (ER-alpha, ER-beta, and possibly other isomers) that, before being incorporated into a TIC, have to be activated by E2. This hormone itself is synthesized, when an initial signal is given, by virtue of an activation of a series of appropriate genes. Via ER, E2 has manifold biological effects. Biological targets of E2 are, inter alia, blood vessel walls [4-8], blood platelets [9], bone [7,10-12], breast cancer cells [13], central nervous system [7,14,15], retinal pigment epithelium [16], synthesis of clotting factors [17].

It is evident that E2 is associated with many biological effects and that many genes must be involved. Consequently, ER must be able to bind to DNA segments, called response elements, in the neighborhood of various genes. The response elements participate, together with other transcription factors, in the formation of TICs that are specific for each gene.

An important problem, currently studied in many laboratories, is to find out which genes are activated in various circumstances. The methods that solve this problem are based on a comparative (differential) approach. A test (target) sample, containing active genes is compared with a control sample in which the genes have not been activated. Using this approach, the active genes are singled out among the multitude of inactive genes. However, the comparisons may reveal the opposite of activation, i.e., downregulation of genes.

Generally, the activity of a gene is characterized by its transcription into mRNAs as the first step leading to the synthesis of specific proteins. Non-activated genes in the control tissue do not produce any corresponding mRNAs. In most methods, the mRNAs prepared from the test and control tissue are each reverse transcribed into the corresponding complementary deoxyribonucleic acid (cDNA), in order to enable a substantial increase of the material for analysis by polymerase chain reaction (PCR) [2]. As most methods do not operate with full mRNA transcripts, but with shorter sequences, the allocation of such sequences to known (or unknown) genes has to be found by advanced computer programs and gene databases.

The methods used for the identification of active genes are sketched below. Included are even methods that have not yet been used for the identification of E2-activated genes. It has to be mentioned that only principles, not technical details are dealt with in this review. Neither the techniques of cloning or of identification of genes by sequencing are described here. The readers who are not familiar with these techniques are advised to consult appropriate textbooks [e.g., [18]]. The dedicated computer programs and databases that are needed for the identification of sequences or genes will not be described here either. These can be found in the references quoted below. It will only be mentioned here that the large databases are GenBank  and Celera .

Activated (expressed) genes can be found by comparison of gene contents in the test and control tissues. There are essentially two approaches for finding activated genes: (i) an individual identification, or (ii) an identification of expression profiles after hybridization to a set of known gene fragments (probes) attached to chips in microarrays.

## Individual identification

This approach means that genes are identified individually, even if several genes can eventually be picked up after cloning. There are several methods that can be used.

### Differential display

Differential display seems to be the technically simplest method. Its name stems from the end-point that is a comparison of a side-by-side display of the test and control preparations by electrophoresis. In its basic form, total RNA of the test and control samples is separately subjected to reverse transcription into cDNA that, in turn, is PCR-amplified using arbitrarily chosen primers. The products are applied to a gel electrophoresis and the band(s) that are specific for one of the preparations are cut from the gel, further amplified by PCR (using the same primers) and eventually sequenced [19].

**Figure 1:**
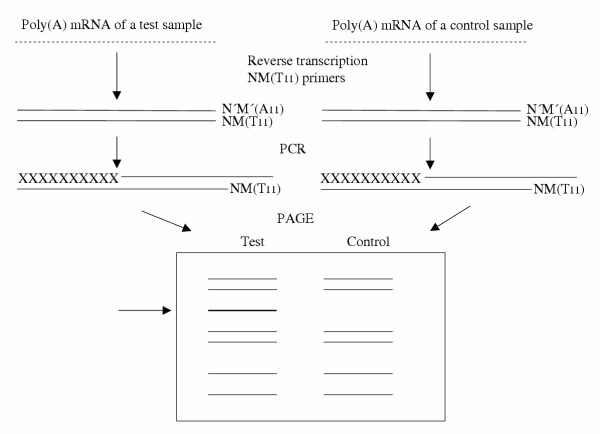
Principle of a differential display. Test and control mRNA are separately reverse transcribed in the presence of anchored oligo(dT) primers containing nucleotides N and M in various combinations (see the text). The same primer and an arbitrary decamer are then used as primers in a PCR. The products are subjected to electrophoresis (PAGE). An additional band (see arrow) in the test sample represents a gene that had not been activated in the control sample. A11 and T11 denote eleven A and T molecules, respectively.

In a more advanced version, mRNAs of the test and control cells are separately reverse transcribed to cDNA (Fig. 1). Each transcription is carried out in the presence of a oligo(dT) primers, directed to the poly(A) tail at the 3' terminus of the mRNA and constructed as 5'(NMT11)3' where N can be guanine (G), adenine (A), thymine (T), or cytosine (C), and M is G, A, or C [20-22]. The primers with G residues are superior to those having one C residue. Those ending in A or T are the least efficient. With use of an arbitrary decamer as the second primer, a PCR is carried out to amplify the transcript in order to obtain a sufficient working material. This is usually done in the presence of a radioactive nucleotide. Other methods are commonly used, such as silver staining. Amplified DNA fragments are separated on a denaturing polyacrylamide gel, the test preparation side by side with the control. Each band differing from those seen in the control electrophoresis is then used for sequencing, subcloning, or as a probe for cDNA library screening. Large amount of results can be obtained depending on the variation in N and M nucleotides. In spite of the basic simplicity of the procedure, the time and workload can be considerable, depending on the number of NM combinations tried.

### Subtractive hybridization with hydroxylapatite separation

**Figure 2:**
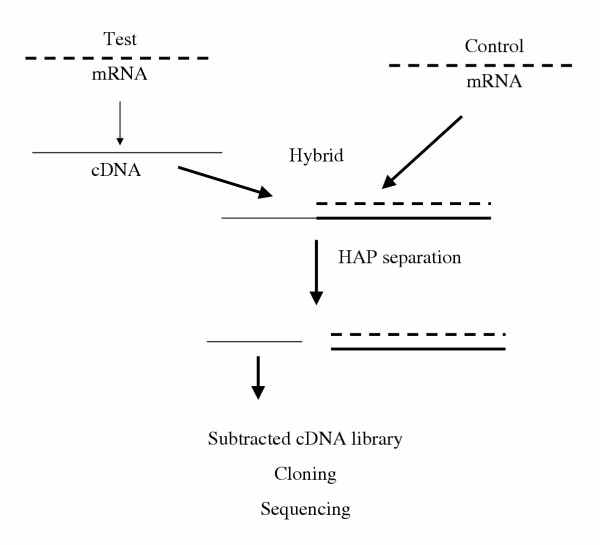
Flow-sheet of subtractive hybridisation with hydroxylapatite separation. Test mRNA is reverse transcribed into a cDNA. This is hybridized with control mRNA. The non-hybridized portion of the single-stranded sequence of test cDNA is separated by chromatography on hydroxylapatite (HAP) and further processed.

The test mRNA is reverse transcribed into cDNA [23]. This is hybridized with the mRNA of the control sample (Fig. 2). A portion of the test cDNA (corresponding to the activated gene) does not find any complementary part in the mRNA of the control sample and remains non-hybridized as a single-stranded cDNA (ss-cDNA). This can be isolated by chromatography on a hydroxylapatite column. The hybridization of the isolated ss-cDNA with control mRNA followed by another chromatography can be repeated to increase the purity of the isolated product [23]. A cDNA library is produced and the subtracted sequence eventually identified. Alternatively, a second hybridization of the isolated ss-cDNA is carried out with the original test mRNA giving rise to a cDNA-mRNA hybrid which, after conversion to double stranded cDNA, is inserted into a vector, a cDNA library is constructed and several specific cDNA clones are isolated, leading to the identification of several genes [24].

In another variant [25], the test and control mRNAs are both reverse transcribed into cDNA. cDNA of the test sample is hybridized with cDNA of the control sample. The non-hybridized part of the test cDNA is a single-stranded DNA that is separated by hydroxylapatite. The single-stranded DNA is cloned into a vector to produce a subtracted library. Clones with a strong hybridization signal to the subtracted probe are selected and sequenced.

### Subtractive suppression hybridization with PCR

**Figure 3:**
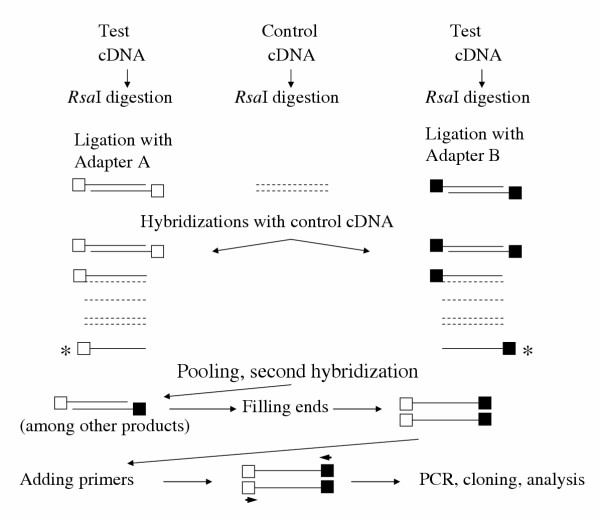
Outline of subtractive suppression hybridisation with PCR. Test cDNA and control cDNA are digested with *Rsa*I. The test cDNA sequences are divided into two halves, one of them being ligated with Adapter A (empty squares), the second one with Adapter B (filled squares). Each half is hybridized with control cDNA. The single-stranded (non-hybridized) sequences of both halves (denoted by asterisks) are annealed in a second hybridization step, primers to the Adapters are added and, after PCR, cloning and gene identification are carried out.

Isolation of a single-stranded test cDNA is not needed in this method. mRNAs of the test and control samples are prepared and each is reverse transcribed into cDNA. Each transcript is digested with the enzyme *Rsa*I to obtain shorter, blunt-ended fragments. The test cDNA is divided into two portions (see Fig. 3). One of them is ligated with Adapter A, the second with adapter B. Each portion is hybridized with an excess of control cDNA. A mixture of hybridization products is formed (Fig. 3). A tiny fraction of cDNA remains unhybridized, single-stranded. This is a fragment that may be called specific, or differentially expressed, or subtracted. It originates from the gene that had been activated. It is absent in the control sample. This specific fragment is bound either to Adapter A or B in the two portions. In the second hybridization, the portions are mixed. After annealing, a small amount of the specific fragment is obtained double-stranded. It contains Adapter A on the one end and Adapter B on the other. After adding primers specific for the Adapters, the ends are filled and the specific fragment is amplified by PCR to make sure that sufficient amounts are available for a further processing. Cloning, sequencing and comparing with a gene database establish the identity of the gene(s) [26,27] [ – "PCR-Select Subtraction kit"]. In contrast to the above methods, the primers for PCR amplification are clearly defined, avoiding thus problems with random primers. This method was used in a number of studies, such as the identification of genes upregulated in rats by E2 and progesterone treatment [28]. A predecessor of this technique is the "representational difference analysis" [29,30].

### Expressed sequence tags (EST)

To describe the EST method, the following example is given. cDNA libraries were prepared by reverse transcription from mRNAs of the tissues to be examined [31]. The libraries were converted to plasmids, transfected into Escherichia coli and plated. Hundreds of clones were picked at random. These were subjected to sequencing, followed by computer matching to known genes listed in the GenBank database. The average length of a sequence was 397 bases; ESTs longer than 150 bases were found to be most useful for similarity searches and mapping.

Subtractive hybridization (see above) was used to isolate the ESTs specific for one of the libraries. For example, a fibroblast cell line cDNA library was hybridized with a hippocampus library; the common sequences were removed and the specific hippocampus sequences remained. Using the EST method, more than 2000 human brain genes were identified [32].

### Serial Analysis of Gene Expression (SAGE)

The SAGE allows serial analysis of gene expression, an analysis of thousands of transcripts. It is based on the assumption that a short nucleotide sequence 10 base pairs (bp) – a tag – contains sufficient information to uniquely identify a transcript. In this respect SAGE differs from the EST approach.

**Figure 4:**
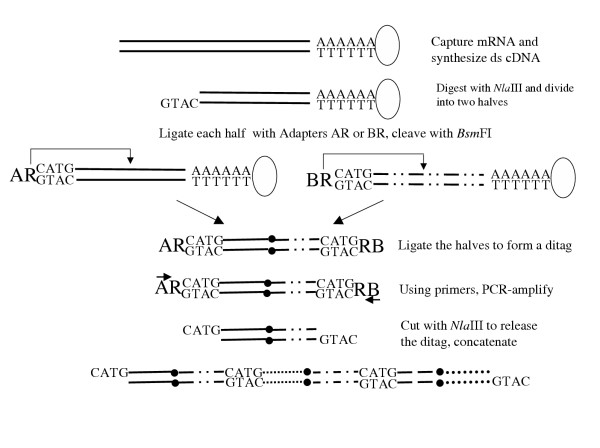
Flow-sheet of SAGE. mRNAs are captured on oligo(dT) magnetic beads (open ovals). Double stranded cDNAs are synthesized. They are digested with *Nla *III. The product is divided into two halves. These are ligated to 40 bp adapters AR and BR, respectively. Both adapters contain a sequence R that is a recognition site for the restriction enzyme *Bsm*FI. This cuts a 14 bp sequence 3' of the site, forming a 10 bp tag. After cleavage with *Bsm*FI, the tags are ligated to form a product containing a ditag (the points of ligation are denoted by filled circles). This is amplified using primers complementary to A and B. The AR and BR adapters are cut away with *Nla *III to release a ditag. These are ligated to form concatemers containing multiple ditags. The concatemers are cloned and sequenced.

The principle of SAGE is as follows: mRNA is reverse transcribed into cDNA with use of a biotinylated primer, the cDNA is cleaved with a restriction endonuclease and the 3' portions are then isolated by binding to streptavidin beads [33]. In another version() (Fig. 4), mRNAs are captured prior to reverse transcription on oligo(dT) magnetic beads. Double stranded cDNAs are synthesized and digested with the restriction endonuclease *Nla*III that cleaves most transcripts at least once. The part attached to the magnetic bead is further processed. The reaction mixture is divided into two portions. The portions are ligated via a restriction site R to an adapter A and B, respectively, each consisting of 40 bp. Taking advantage of the restriction sites R, both portions are cleaved with the restriction enzyme *Bsm*FI in the distance of 14 bp. In this way "tags" are formed. Out of these 14 bp, 4 bp are a non-specific segment GTAC. These tags are blunt-ended with the Klenow fragment of DNA polymerase I. The two separate pools of tags are ligated together via a blunt-end ligation to produce "ditags". The ditags, flanked by the adapters A and B, are amplified by PCR with use of primers for A and B. The adapters are removed by the enzyme *Nla*III and the ditags are concatenated. The resulting concatemers (a series of linked ditags) are cloned into a plasmid vector to create a SAGE library. Individual clones are then sequenced. SAGE is carried out for each sample to be compared.

Thanks to the concatenation, many tags can be detected in a single clone [33]. As each tag is supposed to uniquely identify a transcript, SAGE can generate a comprehensive profile of gene expression. Indeed, many unique transcripts were identified with use of SAGE tags [34]. The method is particularly useful for detecting genes of low level of expression or in rare tissues (e.g., early embryo) [35,36]. In addition, the amount of individual tags provides quantitative estimates of gene expression [37].

Still, the specificity of detection of genes with use of the short tags is not absolute. There are two main problems [38]. The first one is that many SAGE tags have no match to known sequences in databases. These tags may represent so far unidentified genes, but their shortness makes it difficult to characterize the genes. The second problem is that the SAGE tags may find multiple matches in the databases [39,40]. Therefore, attempts have been made to increase the specificity by prolongation of the tags by various methods.

One such method is called GLGI (Generation of Longer cDNA fragments from SAGE tags for Gene Identification) [34,38,40]. The main feature of this method is the use of a SAGE tag as the sense primer for the PCR of a segment of cDNA. An anchored oligo(dT) serves as an antisense primer. In this manner a cDNA "tag" of up to several hundred bases is created. However, this method does not seem to improve the specificity of SAGE because even "non-specific" tags are co-amplified.

Better of seems to be another variant of SAGE, the LongSAGE [41]. This is based on the use of tags 21 bp (out of which 4 represent a restriction site), tags longer than those in SAGE. The prolongation of tags is achieved by the use of the restriction endonuclease *Mme*I. The longer tags increase the power of identification of genes, while not diminishing the sensitivity of SAGE given by the use of PCR and concatenation. Theoretical calculations showed that >99.8% of the 21 bp tags were expected to occur only once in a genome.

SAGE was used for the investigation of differences in gene expression in various health conditions. In the studies of breast tumors [37], global gene expression profiles in breast carcinoma cells were compared with those in normal mammary epithelial cells. The patterns of gene clusters in normal tissue were distinctly different from those of tumors of different stage and histological grade. The most dramatic change occurred at the normal-to-in situ carcinoma transition. This change can be an important marker for an early diagnosis. In another study, several genes regulated by estrogen or tamoxifen were identified in an estrogen-dependent breast cancer cell line. One of them was studied closer. It appeared to play a significant role in estrogen-promoted cell growth [42].

## Gene profiles – microarrays

The DNA microarray analysis is used to identify profiles of expressed genes in a given tissue and time. Thousands of known cDNA sequences or oligonucleotides are imprinted on a solid support, sometimes called a chip (e.g., a microscope slide or a nylon membrane), using application robots. Typically, individual spots are 100–300 micrometers in size and are spaced about the same distance apart [43]. More than 30,000 sequences can be fitted on the surface of a chip. These sequences serve as probes. Alternatively, the probes are synthesized in situ (60-mers) [44]. By hybridization, test (target) sequences (cDNAs or cRNAs) are bound to the cognate probes. The basic approach is the comparison of degree of hybridization in the control and test preparation. There are two basic techniques for the detection of hybridization. The control and test preparations are placed on a single chip, or, separately, on two chips.

**Figure 5:**
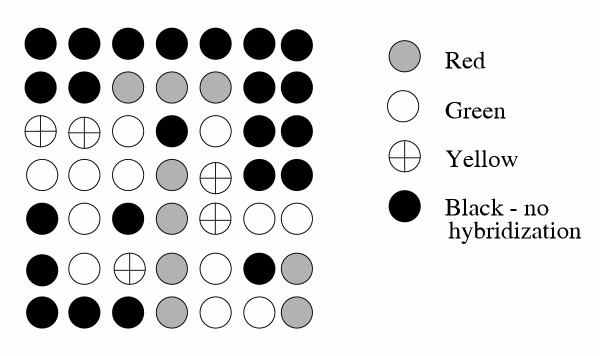
Model of a microarray. In a single-chip technique reverse transcription from mRNAs to cDNAs is separately carried out for the test and control cell preparations. During the transcription one of the fluorescent dyes (e.g., Cy3 – green and Cy5 – red) are incorporated into the cDNAs of each preparation. A mixture of these two preparations is then hybridised to the corresponding gene-representing sequences on a chip. The activated genes of the control sample exhibit green color, those of the test sample provide red spots, equally bound cDNAs can be visualized by yellow spots, no hybridization remains black.

In the single chip technique [18], mRNAs from the control and test cells/tissues are separately reverse transcribed. During the transcription processes two different fluorescent dyes (e.g., Cy3 – green, Cy5 – red) are incorporated into the control and test cDNAs, respectively. The labeled molecules are mixed and hybridized to the cDNA array. There is a competition for each probe on the chip between the control and test mRNAs. The test cDNAs are selectively bound to some probes, the control cDNAs may be bound to other probes. With use of fluorescence scanning it is possible to distinguish the hybrids with control sequences (exhibiting, e.g., green fluorescence) from the hybrids with test sequences (e. g., red) [45]. Alternatively, the dyes may be reversed, and the control and test cDNAs may be labeled with the red and green dye, respectively. The hybrids that arise when the control and test cDNA occur in equal amounts may show a yellow fluorescence. The black spots indicate no hybridization (Fig. 5). One of the commercial companies utilizing this approach is Agilent .

Using a variant of the method [46,47], certain groups of activated genes could be defined as predictors of the clinical outcome of breast cancer. Up to 5000 genes were tested for up-regulation (red) or down-regulation (green) in up to 100 patients with various degrees of disease progression. Correlations of disease grades with gene expression profiles were established, and a strategy was provided to select patients who would benefit from adjuvant therapy.

In the two-chip technique, mRNAs of the test and control tissues/cells is reverse transcribed into a double-stranded cDNA from which a cRNA is prepared. In the course of the cRNA synthesis biotin molecules are incorporated [48]. The control and test cRNAs are separately hybridized to two identical chips. The binding is detected by staining with a fluorescent dye coupled to streptavidin. Signal intensities are used to calculate the relative cRNA abundance for the genes represented on the array. For comparisons of the intensities on both chips advanced computer programs have to be used. A combination of single-chip and two-chip techniques was applied in a study [51] where two chips and two fluorescent dyes were used.

Commercial systems are available from several sources. For example, Affymetrix (GeneChip) [] produce chips by a photolithographic method in which thousands of different oligonucleide probes are synthesized *in situ *on the chip [49]. A compact technique has been introduced by the Febit company [50]. In a single benchtop instrument called Geniom a light-activated oligonucleotide microarray synthesis takes place, as well as addition of biotin-labeled cRNA sample, hybridization and fluorescence detection after incubation with streptavidin-phycoerythrin [50]. Other systems for microarray production, target preparation, hybridization and result evaluation are offered by Amersham Biosciences  and Clondiag Chip Technologies .

As a rule, more than one gene is activated, and a spectrum of genes is discovered either occurring sporadically or in clusters [49]. For example, when a diseased tissue was compared with a healthy one, an expression profile, a disease fingerprint, was identified [49]. In the case of breast tumors, a molecular portrait of each tumor was obtained [52], or, molecular profiling (a set of gene clusters) provided predictions of responses to adjuvant treatment [46,53]. Gene activation in breast cancer cells in the presence of E2 included, apart from the known estrogen-responsive genes, a series of novel genes expressing growth factors and components of the cell cycle, adhesion molecules, enzymes, signaling molecules and transcription factors [48]. Gene expression patterns of breast carcinomas allowed to distinguish tumor subclasses [54]. E2 caused up-regulation of 250 genes in vascular endothelial cells that could be prevented by an inhibitor [55]. In an experimental encephalomyelitis a markedly enhanced gene activation by E2 was noted [56].

Sometimes a technically easier macroarray is used, e.g., on a 96-well plate [57]. Obviously, the choice of gene sequences to be used as probes must be very selective in this case. This approach has been adopted by the SuperArray Bioscience Corporation  offering selected profiles of genes in the macroarray format for various areas (e.g., cancer, cell cycle, cytokine and inflammatory response, etc.).

Quite often the gene identification obtained by an array is confirmed by other methods such as Northern blot analysis [58], or real-time PCR [43,58][]. A negative identification can be achieved by the use of siRNA (small interfering RNA – SuperArray Corp.). siRNAs are short RNA duplexes between 15 to 21 nucleotides in length. Once transfected into cells, a siRNA targets the mRNA containing an identical sequence and degrades it in a catalytic manner. The degraded message is no longer functional in translation (the biosynthesis of protein) and thus in the expression of the corresponding gene. SuperArray Corp. provides a line of validated populations of siRNAs in the form of SureSilencing siRNA kits.

## Conclusions

The methods described above can suit two purposes. The single-gene methods can detect and identify new, previously unknown, genes, whereas microarrays can handle a great number of known genes to establish profiles of their expression.

SAGE seems to have advantages over hybridization-based methods for the studies of gene expression, such as differential display and subtractive hybridization. SAGE is superior to the EST approach in providing high efficiency in identifying the genes that are expressed at low levels and that represent a majority of genes in the human genome [36].

Microarray techniques usually detect activation of a multitude of genes – a gene profile – that differs from the profile in control tissues/cells and thus – in medicine – may have a diagnostic and/or prognostic value. However, the microarray techniques usually require commercially produced chips as well as specialized equipment and advanced, powerful, computing facilities. Thus they are hardly affordable for small or medium-size laboratories unless they have substantial financial resources.

A big question at another level remains so far unanswered: which is the biological "chain of commands" in a given tissue and time resulting in the activation of genes enabling the biosynthesis of cornerstones for gene activation, such as ligands (e.g., E2), receptors (e.g., ER) and other transcription factors, the entire machinery leading to gene activation and expression.
